# Coping with COVID-19: Exploring coping strategies, distress, and post-traumatic growth during the COVID-19 pandemic in Australia

**DOI:** 10.3389/fpsyt.2022.1025767

**Published:** 2022-10-20

**Authors:** Bianca E. Kavanagh, Josephine S. O’Donohue, Melanie M. Ashton, Mojtaba Lotfaliany, Maddy McCallum, Anna L. Wrobel, Sarah Croce, Michael Berk, Lucy Saunders, Jerry Lai, Lesley Berk

**Affiliations:** ^1^School of Medicine, Institute for Mental and Physical Health and Clinical Translation, Barwon Health, Deakin University, Geelong, VIC, Australia; ^2^Orygen, Parkville, VIC, Australia; ^3^Florey Institute for Neuroscience and Mental Health, University of Melbourne, Melbourne, VIC, Australia; ^4^Centre for Youth Mental Health, University of Melbourne, Parkville, VIC, Australia; ^5^Department of Psychiatry, Royal Melbourne Hospital, University of Melbourne, Parkville, VIC, Australia; ^6^Deakin University, eResearch, Deakin eSolutions, Geelong, VIC, Australia; ^7^Intersect Australia, Sydney, NSW, Australia

**Keywords:** COVID-19, mental health, coping, distress, post-traumatic growth

## Abstract

**Objective:**

This study aimed to explore coping strategies, distress, and post-traumatic growth among Australians with and without a history of a mental health diagnosis during the COVID-19 pandemic.

**Materials and methods:**

Australians (*N* = 381) completed an online survey between 4-August 2020 and 25-October-2020. Coping strategies, distress, and post-traumatic growth were ascertained *via* the Brief COPE, Depression Anxiety and Stress Scale (DASS-21), and Post-Traumatic Growth Inventory (PTGI), respectively. Linear regression was conducted to examine the relationship between the Brief COPE, DASS-21, and PTGI, adjusting for sociodemographic factors. Models were conducted separately for those with/without a history of a mental health diagnosis.

**Results:**

Higher distress was found among those with a history of a mental health diagnosis. Significant differences in the types of coping strategies associated with distress and post-traumatic growth were identified between the groups, however, behavioral disengagement and self-blame consistently predicted depression, anxiety, and stress. For those with a history of a mental health diagnosis, positive reframing decreased anxiety. Self-distraction was associated with post-traumatic growth across both groups.

**Conclusion:**

There are important differences in the way people with and without a history of a mental health diagnosis cope with the COVID-19 pandemic.

## Introduction

The COVID-19 pandemic has had considerable global impacts on health and wellbeing (1). The life-threatening burden of physical illness, worry and concern for loved ones, and socio-economic consequences—as well as the effects of numerous and lengthy lockdowns—has been widely felt across the population (2, 3). Pandemic-related restrictions have forced changes to daily life, routine, and social support, increasing distress and isolation (4–7), and have threatened crucial resources within the population (8). These changes, paired with the direct exposure to global threats of life (9), have resulted in trauma-stress reactions (8), comprised of heightened arousal and intrusive re-experiencing of events (10).

The flow-on effects of both the pandemic and associated safety measures have had profound consequences on mental health (3, 11). Within the general population, reviews have documented wide-ranging mental health problems. One global study demonstrated an additional 53.2 million cases of major depressive disorders and 76.2 million cases of anxiety disorders during 2020 due to the pandemic (12). Further, a meta-analysis of 221,970 participants identified pooled prevalence rates for depression, anxiety, distress, and insomnia to be 31.4, 31.9, 41.1, and 37.9% respectively, representing higher than usual rates for these disorders since the beginning of the COVID-19 pandemic (13). In addition to these mental health problems, Hossain et al. (14) noted reports of panic attack, stress and posttraumatic stress, emotional disturbance, irrational anger, impulsivity, sleep disorders, and somatization disorder across the globe. In contrast, Pirkis et al. (15), demonstrated no increase in suicide rates in high- and middle-income countries in the context of the pandemic. Of particular concern, however, is the impact on the mental health of specific groups who may be more vulnerable to experiencing further declines in their mental health, such as those with pre-existing mental health conditions (16).

Individuals with pre-existing mental health conditions may face additional challenges during the COVID-19 pandemic. Yao et al. (17) highlighted that they may face potential relapse or worsening of mental disorder symptoms due to a higher susceptibility to stress, an increased risk of infection, barriers to accessing timely health services, the possibility of treatment being less effective, and difficulty attending regular health appointments due to travel restrictions and lockdowns. O’Connor et al. (18) found that participants with a pre-existing mental health condition were more likely to experience suicidal ideation, higher levels of depression, anxiety, defeat, entrapment, and loneliness, and lower levels of wellbeing compared to those without a mental health condition. Another meta-analysis found that despite an initial increase in mental health symptoms following the World Health Organization’s declaration of a pandemic (i.e., March 2020), these symptoms tended to revert to their pre-pandemic levels by mid-2020 for those with a pre-existing mental health condition, indicating a non-significant change over time (19). The authors noted that this may be due to the naturally occurring recovery of severe mental health symptoms, opportunities for structured routine during lockdowns, and the introduction of new mental health services in some areas. Nonetheless, it is important to consider the factors that may be implicated in distress, as well as what may help with mental health recovery and resilience during this pandemic.

Coping strategies—defined as the cognitions and behaviors that are used to manage stressful situations (20)—may play an important role in overcoming the adversity of the COVID-19 pandemic. Coping may be achieved by using coping actions to alter one’s relationship with the environment (i.e., problem-focused) or by modifying one’s interpretation of the environment (i.e., emotion-focused) (21). Coping strategies may predict mental health outcomes during challenging times. One Australian study found that individuals who experienced high to very high levels of distress, as well as those who had a pre-existing mental disorder, had low resilient coping abilities during the COVID-19 pandemic (22). Somer et al. (23) found that people with pre-existing mental health conditions were more likely to use maladaptive daydreaming as an unhelpful coping strategy during pandemic-related isolation and quarantine. Gurvich et al. (24), in an Australian sample, demonstrated that coping strategies of positive reframing, humor, and acceptance, were associated with better mental health, while self-distraction, self-blame, venting, and behavioral disengagement were related to poorer mental health.

Specific coping strategies may simultaneously preserve mental health and promote post-traumatic growth (i.e., positive psychological transformation following trauma; 25) during the COVID-19 pandemic. Kalaitzaki (26) identified high instances of post-traumatic growth and less frequent use of dysfunctional coping strategies among healthcare workers. Vazquez et al. (27) demonstrated that beliefs about a good world, identification with humanity, and openness to the future was associated with post-traumatic growth. Little research, however, has specifically examined which coping strategies may be implicated in post-traumatic growth and distress during the COVID-19 pandemic, particularly among those with and without a history of a mental health diagnosis within the general population. Further, there are few studies specifically investigating these relationships within Australia—a region that experienced numerous extended restrictions on movement, travel outside the home, and face-to-face social contact throughout the pandemic. These restrictions varied both within and across Australian states and territories.

The aim of the current study was to explore how Australians with and without a history of a mental health diagnosis coped with the impact of the COVID-19 pandemic. Specifically, the study aimed to explore (a) the differences in depression, anxiety, stress, and post-traumatic growth between participants with and without a history of a mental health diagnosis; (b) the coping strategies used by participants with and without a history of a mental health diagnosis; and (c) the relationship between depression, anxiety, stress, post-traumatic growth, and coping strategies among participants with and without a history of a mental health diagnosis.

## Materials and methods

### Participants and procedure

Inclusion criteria for this study were adults (≥18 years), who resided in Australia at the time of survey completion. Data were collected through an online Qualtrics survey (28) between 4-August-2020 and 25-October-2020. The survey was developed and tested by the researchers prior to the commencement of data collection. All participants provided online informed consent prior to participation. Participants were prevented from submitting the survey more than once through the Prevent Multiple Submissions feature of Qualtrics survey software (28), which uses cookies to identify when a survey has already been submitted *via* a particular browser. Participants were able to leave survey items blank and had access to a back button if they wished to return to the previous page of the survey. At the completion of the survey, participants were able to elect if they wanted to enter the draw to win one of ten $50 Coles/Myer gift vouchers and their contact details were collected *via* a parallel survey.

This study was approved by the Barwon Health Human Research Ethics Committee (20/76) and was conducted in accordance with the Good Clinical Practice guidelines.

### Measures

Demographic and lifestyle information, including age, gender, state/territory of residence, country of birth, educational attainment, current employment status, and current living situation was self-reported. Information on whether participants had ever received a mental health diagnosis (yes/no) and if, applicable, the type of diagnosis they had received was also self-reported.

The Brief COPE (29) was utilize to measure strategies used to cope with stressful experiences. The Brief COPE is a 28-item self-report instrument, comprised of 14 subscales of coping strategies, each comprising of two items. These subscales include: self-distraction, active coping, denial, substance use, emotional support, instrumental support, behavioral disengagement, venting, positive reframing, planning, humor, acceptance, religion, and self-blame. Responses are given on a 4-point Likert scale ranging from 0 *(I haven’t been doing this at all)* to 3 *(I have been doing this a lot)*, with higher scores indicating increased utilization of a particular coping strategy. In keeping with the mode of analysis suggested by Carver (29), we examined each subscale separately to examine how it relates to the other variables in the study.

The 21-item version of the Depression Anxiety and Stress Scale (DASS-21) (30) was used to measure the negative emotional states of depression, anxiety, and stress. The DASS-21 is a self-report instrument, which contains three 7-item subscales where participants respond on a 4-point Likert scale from 0 *(did not apply to me at all)* to 3 *(applied to me very much or most of the time)*. This measure has shown strong reliability and validity as a measure of general psychological distress and the independent constructs of stress, anxiety and depression within a large non-clinical sample (31).

Post-traumatic growth was measured with the Post-Traumatic Growth Inventory (PTGI) (32). The PTGI consists of 21 items, responded to on a 6-point Likert Scale ranging from 0 *(I did not experience this change as a result of my crisis)* to *5 (I experience this to a very great degree because of my crisis)*, with higher scores denoting greater post-traumatic growth. The five-factor structure of the PTGI has been replicated in a large sample of Australian adults (33).

### Statistical analysis

Demographic characteristics and scores on the Brief COPE, DASS-21, and PTGI for participants with and without a history of a mental health diagnosis were compared using Student’s *t*-tests and Chi-squared tests. Linear regression models were used to examine the relationships between the Brief COPE subscales as exposures and DASS-21 and PTGI as the outcomes. Models were adjusted for age, sex, education, and location. The effect modification of history of mental health diagnosis on the relationship between coping strategies, DASS-21, and PTGI was checked by adding first-order interaction terms into the model. As strong evidence was found for effect modification, linear regression models were conducted separately for those with and without a history of a mental health diagnosis.

Expected mean changes in DASS-21 and PTGI scores for one-unit increase in score of each coping strategy, after accounting for other coping strategies and potential confounders, were reported along with 95% confidence intervals (CI). Missing data were managed using pairwise deletion as models were fitted on data of participants who had no missing information on the required variables. Analyses were performed using R version 4.0.2 (34).

## Results

A total of 425 survey responses were collected. After removing 44 cases that did not specify mental health diagnosis history, 381 survey responses were included in the analyses (89.6% response rate). Of the 381 participants (*M_*age*_* = 44.2, *SD* = 13.3, range 18.0–81.0 years), 333 (87.4%) reported that they currently lived in Victoria, with 97 (25.7%) participants living in Melbourne and Mitchell shire council localities (subject to more restrictions and lockdowns), and 233 (61.6%) living in non-metropolitan Victoria (where fewer restrictions were in place). Most participants were female (*n* = 320, 84.2%), held a tertiary-level education (*n* = 133, 88.9%), and worked full-time (*n* = 270, 70.9%). A total of 149 (39.1%) participants reported a history of a mental health diagnosis. Differences in living arrangement, whether participants had lost their job due to the COVID-19 pandemic, and general wellbeing and mental health change were identified between those with and without a history of a mental health diagnosis (all *p* < 0.05). Demographic characteristics of the sample are shown in Table 1.

**TABLE 1:** Characteristics of participants by their history of diagnosis for mental disorders.

	Without a history of a mental health diagnosis (*n* = 232)	History of a mental health diagnosis (*n* = 149)	Total (*n* = 381)	*P-value*
Female^$^	197 (84.9%)	123 (83.1%)	320 (84.2%)	0.638
Age^%^				0.011
Mean (SD)	45.6 (12.8)	42.1 (13.3)	44.2 (13.1)	
English as first language^%^				0.011
Yes	202 (89.4%)	141 (96.6%)	343 (92.2%)	
Education				0.094
<High school (year 12 or equivalent)	6 (2.6%)	2 (1.3%)	8 (2.1%)	
High school (year 12 or equivalent)	20 (8.6%)	14 (9.4%)	34 (8.9%)	
Diploma/trade certificate	27 (11.6%)	27 (18.1%)	54 (14.2%)	
Bachelor degree	89 (38.4%)	68 (45.6%)	157 (41.2%)	
Master degree	57 (24.6%)	25 (16.8%)	82 (21.5%)	
PhD/doctorate	33 (14.2%)	13 (8.7%)	46 (12.1%)	
Location^§^				0.078
Interstate	22 (9.6%)	26 (17.4%)	48 (12.7%)	
Metro/Mitchell	62 (27.1%)	35 (23.5%)	97 (25.7%)	
Non-metro	145 (63.3%)	88 (59.1%)	233 (61.6%)	
Employment status				
Works full-time (yes)	165 (71.1%)	105 (70.5%)	270 (70.9%)	0.891
Works casually (yes)	11 (4.7%)	11 (7.4%)	22 (5.8%)	0.281
Student (yes)	23 (9.9%)	18 (12.1%)	41 (10.8%)	0.505
Retired	18 (7.8%)	5 (3.4%)	23 (6.0%)	0.078
Looking for work	5 (2.2%)	7 (4.7%)	12 (3.1%)	0.166
Receiving government benefits	6 (2.6%)	5 (3.4%)	11 (2.9%)	0.662
Lost job due to COVID	1 (0.4%)	5 (3.4%)	6 (1.6%)	0.025
Living arrangement′				0.039
Lives alone	27 (11.8%)	30 (20.4%)	57 (15.2%)	
Lives with family members	192 (84.2%)	108 (73.5%)	300 (80.0%)	
Lives with people outside family	9 (3.9%)	9 (6.1%)	18 (4.8%)	
General wellbeing and mental health change∼				0.016
Declined	127 (55.0%)	103 (69.1%)	230 (60.5%)	
Improved	12 (5.2%)	10 (6.7%)	22 (5.8%)	
Stayed the same	91 (39.4%)	36 (24.2%)	127 (33.4%)	

Values reported as M (SD) or n (%). ^$^Total = 380; ^%^Total = 379; ^§^Total = 378; ′Total = 375; ∼Total = 373.

Coping strategy scores for the total sample and participants with and without a history of a mental health diagnosis are presented in Table 2. Across the sample, the highest mean score was found for acceptance (*M* = 6.4, *SD* = 1.5) and the lowest mean score was denial (*M* = 2.4, *SD* = 0.9), indicating that these strategies were used “a lot” and “a little,” respectively. Aside from acceptance, all remaining coping strategies tended to be used between “a little” and “a lot” by those with and without a history of a mental health diagnosis (*M* = 2.4, *SD* = 0.8 to *M* = 5.6, *SD* = 1.6). Coping strategies of self-distraction, substance use, instrumental support, behavioral disengagement, venting, humor, and self-blame were used significantly more frequently by participants with a history of a mental health diagnosis, compared to those without (all *p* < 0.05).

**TABLE 2:** Mean (SD) scores for the Brief COPE, DASS-21, and PGTI among the total sample and those with and without history of mental disorder.

	Without a history of a mental health diagnosis *M* (SD)	History of a mental health diagnosis *M* (SD)	Total *M* (SD)	*P-value*
**Brief COPE**				
Self-distraction^$^	5.2 (1.6)	5.6 (1.6)	5.3 (1.6)	0.005
Active coping^%^	5.3 (1.6)	5.2 (1.6)	5.3 (1.6)	0.964
Denial^§^	2.4 (0.8)	2.5 (0.9)	2.4 (0.9)	0.261
Substance use^§^	2.9 (1.5)	3.5 (1.8)	3.1 (1.6)	0.001
Emotional support^%^	4.8 (1.6)	5.0 (1.6)	4.9 (1.6)	0.180
Instrumental support′	3.8 (1.5)	4.3 (1.7)	4.0 (1.6)	0.002
Behavioral disengagement^%^	2.7 (1.1)	3.1 (1.4)	2.8 (1.2)	0.005
Venting′	3.9 (1.3)	4.2 (1.4)	4.1 (1.4)	0.037
Positive reframing′	5.1 (1.7)	5.1 (1.6)	5.1 (1.7)	0.998
Planning∼	5.0 (1.6)	5.0 (1.7)	5.0 (1.7)	0.991
Humor^$^	3.8 (1.5)	4.4 (1.8)	4.0 (1.6)	0.001
Acceptance^$^	6.4 (1.5)	6.3 (1.5)	6.4 (1.5)	0.293
Religion^§^	3.1 (1.7)	3.1 (1.6)	3.1 (1.7)	0.913
Self-blame^%^	3.1 (1.4)	4.0 (1.8)	3.4 (1.6)	<0.001
**DASS-21**				
Depression^$^	10.1 (8.6)	14.3 (9.9)	11.7 (9.3)	<0.001
Anxiety^$^	5.0 (5.8)	8.7 (7.7)	6.4 (6.8)	<0.001
Stress^$^	13.2 (8.4)	17.4 (8.7)	14.8 (8.7)	<0.001
PTGI total score^∧^	31.2 (21.3)	31.1 (21.2)	31.1 (21.3)	0.971

^$^Total, 367; ^%^Total, 366; ^§^Total, 365; ′Total, 364; ∼Total, 363; ^∧^Total, 330 for whole sample.

DASS-21, Depression Anxiety Stress Scale; SD, Standard Deviation; PTGI, Post-Traumatic Growth Index.

Test for effect modification revealed strong evidence for interaction between mental health diagnosis and DASS-21, Brief COPE, and PTGI (*p*-values for interaction < 0.05). Therefore, we conducted separate linear regression models for those with and without a history of a mental health diagnosis. Mean scores on the DASS-21 indicated there were severe levels of depression (*M* = 11.7, *SD* = 9.3) and stress (*M* = 14.8, *SD* = 8.7), and moderate levels of anxiety (*M* = 6.4, *SD* = 6.8), across the total sample. Participants with a history of a mental health diagnosis reported significantly higher (i.e., poorer) scores across each of the subscales of the DASS-21, compared to those without a history of a mental health diagnosis (all *p* < 0.001). No significant differences were found in PTGI scores between participants with and without a history of a mental health diagnosis (*p* = 0.971). DASS-21 and PTGI scores for the total sample and for participants with and without a history of a mental health diagnosis are also displayed in Table 2.

Table 3 demonstrates the results from the adjusted linear regression models showing associations between the Brief COPE, DASS-21, and PTGI scores for the total sample. After adjusting for age, sex, education, and participant location, a one-unit increase in acceptance and religion was associated with a 0.76 and 0.43 decrease in reported depression on the DASS-21. Coping strategies of self-distraction, substance use, instrumental support, behavioral disengagement, planning, and self-blame were each associated with an increase in depression by 0.43, 0.46, 0.93, 3.30, 0.55, and 1.59 units respectively, for each one-point increase in these strategies. On the anxiety subscale, for a one-point increase in behavioral disengagement and self-blame, these coping strategies were associated with a 0.76 and 1.47 unit increase in reported anxiety. On the PGTI, a one-point increase in self-distraction, denial, and positive reframing was associated with 2.23, 3.55, and 3.30 unit increase in post-traumatic growth. Scores on the remaining coping strategies and post-traumatic growth items tended to vary across the subscales (see Table 3), but these results were not significant.

**TABLE 3:** Adjusted associations between the Brief COPE, DASS-21, and PTGI for the whole sample.

	DASS-21^$^									PTGI total^%^		
	Depression			Anxiety			Stress					
Self-distraction	0.43	0.01, 0.85	0.044	–0.01	–0.40, 0.38	0.957	0.36	–0.08, 0.80	0.110	2.23	0.87, 3.59	0.001
Active coping	–0.50	–1.06, 0.05	0.075	0.18	–0.33, 0.69	0.485	–0.35	–0.93, 0.23	0.237	0.38	–1.43, 2.19	0.682
Denial	–0.21	–1.11, 0.68	0.637	0.47	–0.35, 1.29	0.263	–0.57	–1.51, 0.37	0.232	3.55	0.61, 6.50	0.018
Substance use	0.46	0.05, 0.88	0.030	0.35	–0.04, 0.73	0.078	0.26	–0.18, 0.70	0.245	0.76	–0.65, 2.17	0.289
Emotional support	–0.51	–1.08, 0.06	0.081	–0.07	–0.59, 0.46	0.802	–0.58	–1.18, 0.02	0.056	1.75	–0.08, 3.59	0.061
Instrumental support	0.93	0.33, 1.54	0.003	0.44	–0.12, 0.99	0.121	0.74	0.11, 1.38	0.021	–0.31	–2.23, 1.62	0.754
Behavioral disengagement	3.30	2.63, 3.96	<0.001	0.76	0.15, 1.37	0.014	1.60	0.90, 2.29	<0.001	–0.55	–2.77, 1.67	0.627
Venting	0.46	–0.09, 1.01	0.098	0.32	–0.19, 0.82	0.217	0.85	0.28, 1.43	0.004	0.00	–1.78, 1.79	0.997
Positive reframing	–0.45	–0.95, 0.04	0.074	–0.59	–1.04, –0.13	0.012	–0.26	–0.78, 0.25	0.317	3.30	1.70, 4.91	< 0.001
Planning	0.55	0.00, 1.10	0.049	0.05	–0.45, 0.55	0.853	0.53	–0.05, 1.10	0.072	1.02	–0.73, 2.77	0.250
Humor	–0.41	–0.84, 0.02	0.063	0.16	–0.24, 0.55	0.441	–0.22	–0.68, 0.23	0.334	–0.90	–2.36, 0.55	0.224
Acceptance	–0.76	–1.31, –0.21	0.007	–0.42	–0.92, 0.08	0.102	–1.04	–1.62, –0.47	<0.001	–1.11	–2.93, 0.72	0.234
Religion	–0.43	–0.83, –0.03	0.037	0.05	–0.32, 0.42	0.783	–0.26	–0.68, 0.16	0.226	1.26	–0.05, 2.57	0.059
Self-blame	1.59	1.09, 2.08	<0.001	1.47	1.02, 1.93	<0.001	2.10	1.58, 2.61	<0.001	–0.87	–2.49, 0.76	0.297

Values reported as estimates with 95% confidence intervals and *p*-values.

^$^Total = 341; %317; DASS-21, Depression Anxiety Stress Scale; PTGI, Post-Traumatic Growth Index.

The results of the adjusted regression models for association between the Brief COPE, DASS-21, and PTGI among those with and without a history of a mental health diagnosis are shown in Table 4. For participants without a history of a mental health diagnosis, denial, behavioral disengagement, venting, planning, and self-blame were associated with a 1.23, 2.80, 0.78, 1.02, 1.78 unit increase in depression, respectively, for each one-point increase in these strategies. For a one-point increase in denial and self-blame, there was a 1.37 and 1.18 unit increase in anxiety, respectively. For the stress subscale, a one-point increase in behavioral disengagement, venting, and self-blame was associated with a 2.15, 1.17, 1.81 unit increase in stress, while emotional support and acceptance were associated with a 0.87 and 1.10 decrease in stress, respectively, for each one-point increase in these strategies. For the PTGI, a one-point increase in self-distraction, emotional support, positive reframing was associated with a 2.37, 2.46, 3.98 unit increase in post-traumatic growth, respectively. The direction of the relationships between the other coping strategies and post-traumatic growth were varied and not significant (see Table 4).

**TABLE 4:** Adjusted associations between Brief COPE, DASS-21, and PTGI scores among those with and without history of diagnosis for mental disorders.

	DASS-21^$^									PTGI TOTAL^%^		
	Depression			Anxiety			Stress					
**Without a history of a mental health diagnosis**												
Self-distraction	0.48	–0.04, 1.00	0.068	–0.20	–0.68, 0.27	0.397	0.07	–0.52, 0.66	0.817	2.37	0.58, 4.17	0.010
Active coping	–0.51	–1.17, 0.15	0.132	0.06	–0.54, 0.67	0.842	–0.31	–1.06, 0.45	0.426	0.84	–1.48, 3.16	0.475
Denial	1.23	0.04, 2.42	0.044	1.37	0.27, 2.46	0.015	–0.19	–1.56, 1.18	0.780	3.58	–0.53, 7.69	0.088
Substance use	0.27	–0.30, 0.84	0.354	0.49	–0.04, 1.01	0.069	0.45	–0.21, 1.10	0.183	–0.16	–2.20, 1.88	0.875
Emotional support	–0.63	–1.31, 0.05	0.071	–0.16	–0.78, 0.47	0.617	–0.87	–1.65, –0.08	0.031	2.46	0.11, 4.80	0.040
Instrumental support	0.35	–0.43, 1.12	0.377	0.45	–0.26, 1.15	0.215	0.52	–0.37, 1.40	0.250	–0.88	–3.52, 1.77	0.514
Behavioral disengagement	2.80	1.87, 3.73	<0.001	0.12	–0.73, 0.97	0.778	2.15	1.08, 3.22	<0.001	2.51	–0.67, 5.69	0.121
Venting	0.78	0.10, 1.45	0.024	0.60	–0.02, 1.21	0.056	1.17	0.40, 1.94	0.003	–0.92	–3.24, 1.39	0.432
Positive reframing	–0.52	–1.13, 0.08	0.091	–0.45	–1.01, 0.10	0.110	–0.09	–0.79, 0.61	0.800	3.98	1.86, 6.11	< 0.001
Planning	1.02	0.32, 1.71	0.004	0.32	–0.32, 0.95	0.325	0.66	–0.13, 1.45	0.102	0.04	–2.31, 2.38	0.976
Humor	–0.24	–0.82, 0.34	0.422	0.03	–0.50, 0.56	0.906	–0.05	–0.72, 0.62	0.882	–0.88	–2.94, 1.18	0.400
Acceptance	–0.64	–1.30, 0.01	0.053	–0.40	–1.00, 0.20	0.193	–1.10	–1.85, –0.35	0.004	–0.80	–3.07, 1.47	0.486
Religion	–0.45	–0.94, 0.04	0.073	–0.09	–0.55, 0.36	0.679	–0.22	–0.79, 0.34	0.438	1.03	–0.63, 2.69	0.222
Self-blame	1.78	1.08, 2.49	<0.001	1.18	0.53, 1.82	<0.001	1.81	1.00, 2.61	<0.001	–1.08	–3.46, 1.30	0.373
**History of a mental health diagnosis**												
Self-distraction	–0.04	–0.79, 0.72	0.921	0.16	–0.58, 0.91	0.664	0.42	–0.29, 1.14	0.245	3.45	0.91, 5.98	0.008
Active coping	–0.27	–1.29, 0.74	0.593	0.58	–0.42, 1.58	0.254	–0.19	–1.15, 0.78	0.702	–0.13	–3.48, 3.22	0.939
Denial	–1.45	–2.95, 0.05	0.057	0.11	–1.37, 1.58	0.886	–0.19	–1.61, 1.23	0.795	4.05	–1.03, 9.13	0.117
Substance use	0.41	–0.25, 1.08	0.221	0.35	–0.31, 1.00	0.298	0.01	–0.62, 0.64	0.985	2.26	–0.06, 4.59	0.056
Emotional support	–0.46	–1.53, 0.61	0.393	0.31	–0.74, 1.37	0.559	0.20	–0.82, 1.21	0.699	0.25	–3.28, 3.78	0.889
Instrumental support	1.28	0.25, 2.31	0.016	–0.03	–1.05, 0.98	0.946	0.56	–0.42, 1.54	0.258	1.32	–1.99, 4.64	0.429
Behavioral disengagement	3.43	2.37, 4.50	< 0.001	0.86	–0.19, 1.91	0.108	0.98	–0.03, 1.99	0.057	–2.73	–6.67, 1.22	0.173
Venting	0.22	–0.75, 1.20	0.651	–0.09	–1.06, 0.88	0.854	–0.03	–0.95, 0.90	0.957	0.35	–2.94, 3.65	0.833
Positive reframing	–0.29	–1.17, 0.59	0.515	–1.01	–1.88, –0.14	0.024	–0.44	–1.28, 0.39	0.296	1.90	–0.94, 4.74	0.188
Planning	0.02	–0.91, 0.94	0.972	–0.37	–1.28, 0.54	0.426	0.17	–0.71, 1.04	0.709	1.57	–1.48, 4.61	0.310
Humor	–0.81	–1.51, –0.11	0.025	0.35	–0.35, 1.04	0.324	–0.58	–1.24, 0.09	0.088	0.07	–2.37, 2.50	0.955
Acceptance	–0.77	–1.79, 0.26	0.140	–0.71	–1.72, 0.30	0.166	–0.66	–1.63, 0.31	0.182	–1.86	–5.51, 1.79	0.315
Religion	–0.19	–0.92, 0.54	0.611	0.27	–0.44, 0.99	0.450	–0.13	–0.82, 0.56	0.712	0.72	–1.79, 3.24	0.570
Self-blame	1.74	0.92, 2.56	<0.001	1.97	1.16, 2.78	<0.001	2.74	1.96, 3.52	<0.001	–0.58	–3.43, 2.28	0.690

Values reported as estimates with 95% confidence intervals and *p*-values.

^$^Total, 341; ^%^317; DASS-21, Depression Anxiety Stress Scale; PTGI, Post-Traumatic Growth Index.

For participants with a history of a mental health diagnosis, a one-point increase in instrumental support, behavioral disengagement, and self-blame was associated with a 1.28, 3.43, 1.74 unit decrease in depression, while a one-point increase in humor was associated with a 0.81 unit decrease in depression. For the anxiety scale, and a one-point increase in self-blame was associated with a 1.97-unit increase, and a one-point increase in positive reframing was associated with a 1.01-unit decrease. A one-point increase in self-blame was associated with 2.74 unit increase in stress. On the PTGI, a one-point increase in self-distraction was associated with a 3.45 unit increase in post-traumatic growth. Associations between the remaining coping strategies and PTGI were not significant and tended to vary in direction (see Table 4).

## Discussion

This study investigated coping strategies, distress, and post-traumatic growth among Australians during the COVID-19 pandemic. There were severe levels of depression and stress, and moderate levels of anxiety across the sample. Participants with a history of a mental health diagnosis experienced higher levels of depression, anxiety, and stress, but there were no significant differences in post-traumatic growth between those with and without a history of a mental health diagnosis. Across both groups, self-blame consistently predicted higher levels of depression, anxiety, and stress; behavioral disengagement was associated with higher depression; and self-distraction was positively associated with post-traumatic growth. Other coping strategies differently predicted distress and post-traumatic growth for those with and without a history of a mental health diagnosis.

Our findings align with previous research showing high levels of distress among the Australian population during the COVID-19 pandemic (22, 24, 35). This coincides with the finding that approximately 60% of participants in the current study reported that their wellbeing and mental health had declined since the beginning of the COVID-19 pandemic. Most participants in the current study resided in Victoria, which experienced repeated and extended lockdowns (36). At the time of data collection, metropolitan Victoria was under Stage 4 restrictions, which included a curfew between 8 p.m. and 5 a.m., a 5 km travel restriction, 1 h of exercise per day, and permission to leave home only for exercise, essentials shopping, approved work, and providing care (37); non-metropolitan Victoria was under stage 3 restrictions, which included permission to leave home only for exercise, essentials shopping, approved work, and providing care (37). However, it is unclear whether these restrictions or other pandemic-related challenges contributed to the impact on wellbeing. Although no specifically comparable data are available, research has shown greater distress among Victorians during times of lockdown than states that were not in lockdown (38). Further, participants with a history of a mental health diagnosis experienced greater distress than those without a history of a mental health diagnosis, supporting the findings put forward by O’Connor et al. (18) and Sampogna et al. (39). It is noteworthy, however, that most participants within the current study resided in non-metropolitan areas of Victoria, which—apart from Mitchell Shire—were subject to less-stringent lockdown measures than metropolitan Melbourne. This, paired with the fact that few participants had reported job loss due to COVID-19, and that most participants were employed full-time and lived with family, may have mitigated even higher levels of distress and changes to wellbeing and mental health due to financial or social concerns over this time, as is seen in other research (i.e., 35).

Although there is disagreement concerning the conceptualization of coping strategies, Krzysztof (40) put forward positive emotional coping (i.e., the regulation of emotional responses to a problem through positive emotion) as a core coping style that is associated with better mental health outcomes. In the current study, humor and positive reframing were predictive of decreased depression and anxiety, respectively, for those with a history of a mental health diagnosis. Humor refers to making jokes about/fun of the situation, while positive reframing denotes viewing the situation in different light to make it seem more positive/looking for something good within the situation (29). Acceptance—accepting the reality of/learning to live with the situation (29)—and emotional support—receiving emotional support/comfort and understanding from others—significantly lowered stress for those without a history of a mental health diagnosis. With the exception of emotional support, these findings are akin to Gurvich et al. (24), who also found humor, positive reframing, and acceptance lowered distress during COVID-19 in Australia. Research from Saudi Arabia, however, found that less use of these strategies did not predict distress (41)—potentially suggesting that cultural differences or variations in societal responses to the pandemic may be at play, methodological differences notwithstanding.

The use of different coping strategies by those with and without a history of a mental health diagnosis is notable. Reasons for this disparity may potentially be a result of participants with a history of a mental health diagnosis having learnt and benefited from specific coping strategies in response to even pre-pandemic times of distress. Particular psychological interventions—such as cognitive behavioral therapy—emphasize reframing cognitions, which entails identifying and changing the perception of a stressor to be more balanced or positive (42), while humor has been found to be useful during situations in which people have low control (40), such as during the pandemic. These differences may support the notion that individuals tend to engage in coping “styles” that are stable across time and situations (43), suggesting that for those with a history of a mental health diagnosis, particular strategies that may have been employed during pervious crises have also been used during the pandemic. Particular cognitive schemas and personality traits that are implicated in the development of mental disorders (e.g., neuroticism) may also prompt engagement in particular coping strategies (43). These individual differences may play a role in the distinctions seen between those with and without a history of a mental health diagnosis, however, the measurement of personality was beyond the scope of the current study.

The finding that, across both groups, self-blame consistently predicted higher levels of depression, anxiety, and stress, and that behavioral disengagement was associated with higher depression, is concordant with other research in the area. Gurvich et al. (24), found that behavioral disengagement, self-blame, venting, instrumental support, and self-distraction were predictive of poor mental health. The present study found that venting (i.e., expressing negative/unpleasant feelings), planning (i.e., coming up with strategies/steps to take), and denial (i.e., refusing to believe what has happened) were associated with increased distress for those without a history of a mental health diagnosis, while instrumental support (i.e., attempting or receiving advice from others) predicted increased levels of depression for those with a history of a mental health diagnosis. Although most of these strategies are largely considered to be “ineffective” or “less useful” strategies (40), Lazarus and Folkman (20) contend that strategies cannot be judged to be effective or ineffective independent of the context that they are used in. Nonetheless, the association between such strategies and poor mental health has been documented in other research on COVID-19 (e.g., 44) and Severe Acute Respiratory Syndrome (e.g., 45, 46), suggesting that these strategies may not be conducive to coping well specifically during times of pandemic stress.

Our results indicated that self-distraction was associated with greater post-traumatic growth across both groups. This finding is noteworthy given that self-distraction was found to be predictive of depression for the whole group, but no differences were evident when the mental health diagnosis groups were examined separately. Self-distraction refers to the ability to engage in work or other activities to take one’s mind off things/think about a situation less (43). Thus, regardless of one’s history of a mental health diagnosis, individuals engaged in self-distracting activities during the COVID-19 pandemic, and this led to high instances of both depression and post-traumatic growth. Sampogna et al. (39), in a large Italian sample, found that self-distraction was the only coping strategy that predicted both depression and anxiety, and that self-distraction also negatively predicted resilience. However, the authors did not examine the impact of self-distraction on post-traumatic growth. Interestingly, in the current study, self-distraction was the only coping strategy associated with post-traumatic growth among those with a history of a mental health diagnosis. Emotional support and positive reframing, however, were predictive of post-traumatic growth for individuals without a history of a mental health diagnosis. Although we found differences in the relationship between coping strategies and post-traumatic growth between those with and without a history of a mental disorder, our findings are largely consistent with the literature suggesting that the recognition of an event as traumatic appears to be a condition of post-traumatic growth (47, 48), and that the strength of this association coincides with the magnitude of the event (49). Vazquez et al. (27) found that post-traumatic symptoms were associated with post-traumatic growth during the first-wave of COVID-19 (i.e., April 2020) in Spain. The authors also found that beliefs about a good world, identification with humanity, and openness to the future was indicative of greater post-traumatic growth, and these characteristics may align with elements of emotional support and positive reframing seen to be beneficial to post-traumatic growth within the current study. Menculini et al. (50), found that appreciation for life and personal strength were the most highly rated dimensions of post-traumatic growth during the first-wave of COVID-19 in Italy and scores on these dimensions did not differ according to whether or not participants had a pre-existing mental disorder. However, only 15% of the Menculini et al. (50) sample reported significant growth in at least one dimension of a short-form version of the PGTI, suggesting that differences in post-traumatic growth across countries may be a product of dissimilar social contexts during the COVID-19 pandemic.

This study has several strengths, including the use of well-validated measures to assess coping strategies, distress, and post-traumatic growth. The timing of data collection (i.e., within one of the most restrictive and lengthy lockdowns experienced in Victoria) was also able to provide timely insights into the experiences of the public. Several limitations are worth noting, however. Despite attempting to obtain a representative Australian sample, most participants resided in Victoria—a region which may have a unique experience of the impact of COVID-19 due to region-specific restrictions. In addition, over 60% of our sample lived in non-metropolitan Victoria, and this proportion is approximately double to what is found within the general population. Most of our sample was highly educated and female, and accordingly, results may not be generalizable to the wider population, especially considering that research has shown that females attain greater post-traumatic growth than males (26). Data were collected *via* self-report at one time-point, which may have introduced biases into the results. Heterogeneity and low numbers precluded a nuanced analysis of differences between individuals with different types of diagnoses. Finally, data were analyzed cross-sectionally and it is unknown whether experiences of coping, distress, and post-traumatic growth changed as the COVID-19 pandemic continued over time.

The current findings give rise to a number of practical implications that may be considered during the COVID-19 pandemic or other significant global health events, including:

• Consideration of the public’s distress in emergency public health messaging through the adoption of a psychosocial lens that is sensitive to contextual differences (51).
• Inclusion of mental health support as part of response efforts (51).
• Provision of additional or intensive support to at-risk groups within the population, including those with a history of a mental health diagnosis.
• Encouragement of the public to adopt positive emotional coping strategies (i.e., using positive emotions to regulate emotional responses) to decrease distress.
• Recognition of protective factors within the community to develop tailored interventions to prevent distress and mitigate the development of mental disorders on a large scale (50).
• Encouragement of specific ways of coping (e.g., self-distraction) to enhance post-traumatic growth in the pandemic context.
• Provision of additional research to further investigate the utility of engaging in specific coping strategies during the pandemic.

## Conclusion

This study found that considerable distress was present among the general—mainly Victorian—population during the COVID-19 pandemic, and that those with a history of a mental health diagnosis experienced higher levels of distress than those without a history of a mental health diagnosis. Our findings indicated that there were important differences in the way people with and without a history of a mental health diagnosis coped with the COVID-19 pandemic, but that positive emotional coping strategies decreased distress across both groups, while strategies that have traditionally been considered as “ineffective” or “less useful” were mainly associated with greater distress. Self-distraction was associated with post-traumatic growth across both groups, and those without a history of a mental health diagnosis additionally engaged in emotional support and positive reframing to increase post-traumatic growth. Given that the COVID-19 pandemic is ongoing, the findings of this study may be helpful to address concerns for the general wellbeing and mental health of the Australian public.

## Data availability statement

The raw data supporting the conclusions of this article will be made available by the authors, without undue reservation.

## Ethics statement

The studies involving human participants were reviewed and approved by the Barwon Health Human Research Ethics Committee. The patients/participants provided their written informed consent to participate in this study.

## Author contributions

LB, MM, AW, MA, and JL designed the survey. LB, LS, MM, AW, MA, and SC assisted with data collection. ML performed the statistical analyses. All authors edited and approved the final version of this manuscript.
